# Associations between social support and physical activity among postpartum women: a cohort study

**DOI:** 10.1093/eurpub/ckac131.440

**Published:** 2022-10-25

**Authors:** KE Bennetter, CW Waage, KR Richardsen, AK Jenum, NK Vøllestad, HS Robinson

**Affiliations:** 1 University of Oslo, Institute of Health and Society, Oslo, Norway; 2 Oslo Metropolitan University, Faculty of Health Science, Oslo, Norway

## Abstract

**Background:**

Moderate-to-vigorous physical activity (MVPA) enhances postpartum women’s health, and social support is associated with higher self-reported physical activity (PA) postpartum. It is unknown if this association exist across ethnic groups. Our research questions are: are overall family or friends’ support associated with objectively recorded MVPA postpartum, 2) are specific types of family or friends’ support associated with MVPA and 3) does the association differ across ethnic groups?

**Methods:**

We used data from 662 women participating in the STORK Groruddalen cohort study (2008-2010). MVPA in bouts ≥ 10 minutes was recorded by SenseWear ArmbandTM Pro3 14 weeks postpartum. Family and friends’ support was measured by the Social Support for Exercise Scale. We used single items and mean score for family (6 items) and friends’ (6-items) support in separate linear regression models, and adjusted for age, ethnicity, education, parity, weeks since birth and body mass index. We tested for interactions between social support and ethnicity. Analyses were performed on complete cases and imputed data due to missing MVPA data.

**Results:**

Based on imputed data we observed an association between family support and MVPA (β = 4.0, 95% CI, 0.18 to 7.74, p = 0.040). Women reporting high family support on two specific items spent 9 MVPA minutes/day more than women reporting low support (‘discuss PA': β = 8.6, 95% CI: 0.37 to 16.87 and ‘co-participation': β = 8.8, 95% CI: 1.79 to 15.86). Associations were not modified by ethnicity. No statistically significant association between friends’ support and MVPA was observed. Similar results were found in complete case analyses, with few exceptions.

**Conclusions:**

Overall family support and specific form of family support (i.e., PA discussion and co-participation) were associated with MVPA postpartum across ethnic groups. Friends’ support was not associated with MVPA.

**Key messages:**

• Postpartum health may be improved across ethnic groups through increased PA facilitated through overall family support for PA and specifically through PA discussions and co-participation from family.

• Friends’ support for PA was not associated with PA.

